# One size does not fit all: advanced practice provider considerations for the antimicrobial steward

**DOI:** 10.1017/ash.2023.199

**Published:** 2023-08-18

**Authors:** Kyleen E. Swords, Gina M. Weddle, Joshua C. Herigon, Paula D. Stering, Matthew S.L. Lee

**Affiliations:** 1 Division of Infectious Diseases, Beth Israel Deaconess Medical Center, Boston, MA, USA; 2 Division of Infectious Diseases, Children’s Mercy Kansas City, Kansas City, MO, USA

## Abstract

Advanced practice providers are a diverse and established group of antimicrobial prescribers in both ambulatory and inpatient settings. We outline important considerations for antimicrobial stewardship programs and stewards to consider when engaging this important group of providers.

## Introduction

Advanced practice provider (APP) is a term that indicates a group of healthcare providers, generally referring to physician assistants (PAs) and nurse practitioners (NPs), but also includes clinical nurse specialists, certified registered nurse anesthetists (CRNAs), and certified nurse midwives (CNMs).^1^ APPs provide evidence-based care throughout the lifespan of patients and across many different disciplines and practice settings, making them an important group to consider for antimicrobial stewardship interventions and utilization as stewards. We want to emphasize and highlight the diversity of the term “Advanced Practice Provider”; however, we have chosen to focus our review on PAs and NPs given almost all infectious diseases (ID), and antimicrobial stewardship-related literature has been published on these two provider groups.

## Background and diversity of APPs

In 2022, there were a reported 335,000 NPs and 159,000 PAs in the United States.^2,3^ Both NPs and PAs require graduate-level degrees (Master’s degree for PA; Master’s or Doctoral degree for NPs) and national board certification prior to obtaining state licensure; however, there are key differences. NP candidates have already obtained registered nurse (RN) licensure, and programs typically offer multiple training tracks, including, but not limited to, Family (FNP), Pediatric (PNP-PC), or Adult-Geriatric (AGNP) tracks, as well as Acute Care (ANP), Women’s Health and Psych-Mental Health. Each track focuses on providing tailored education and clinical experience to the population foci in which a NP seeks certification. In contrast, PA training programs tend to have a singular track modeled on medical school curricula with a broad range of didactics and clinical rotations.^3^ While curricula regarding ID and antimicrobials vary between APP training programs, one study found 95% of NP programs had lectures on antimicrobials, but most programs had less than 10 hours of total content.^4^

Historically, APPs were utilized as a solution to the primary care physician shortage with a positive impact on patient outcomes and patients’ satisfaction in their care. However, in the setting of physician trainee work hour restrictions, there has been evolution of practice scope with increasing inpatient involvement.^5,6^ Currently, almost 10% of NPs have acute care certification with the majority working in hospital-based inpatient settings.^7^ Over 35% of PAs currently report working in hospital-based settings with the highest frequency in surgical and internal medicine sub-specialties.^3^ A study of Veteran’s Health Administration data showed over 50% of inpatient medical services now utilize APPs.^8^ Although it is not a requirement for employment or clinical practice, APP residencies and fellowships are becoming more popular, providing APPs with more hands-on and specialized training in certain disciplines, including emergency medicine, surgical sub-specialties, hospital medicine, and ID.^9–11^

## What we know about APP antimicrobial usage

There are limitations about APP antimicrobial utilization, as most studies have been done in the ambulatory setting with a focus on NPs. Studies have found that APPs now provide over 25% of outpatient antibiotic prescriptions in the United States.^12,13^ Comparison between APP and physician prescribing practices remains limited to the ambulatory setting, where antimicrobial stewardship interventions are not common and have varied results.^14^ In these settings, several studies showed a higher rate of antibiotic prescriptions from APPs compared to physicians, while others showed no difference.^12,15–20^ A more recent study in the VA healthcare system showed a decrease in outpatient antibiotic prescriptions from APP prescribers over time, even after independent prescribing privileges were granted at the VA.^21^

As noted, limited literature exists about APP prescribing for both non-antimicrobials and antimicrobials in the inpatient setting. One study of a multi-disciplinary orthopedic surgery teams at an academic medical center found that NPs and PAs were responsible for a similar amount of discharge opioid prescriptions as post-graduate years 1–3 residents; however, no studies have been published comparing inpatient antimicrobial prescriptions between APPs and other prescribers.^22^

There are differences between APPs practicing in ambulatory settings, where there might be a higher level of autonomy, versus APPs working in inpatient settings. APPs on inpatient care teams may work as the primary hospitalist or as part of care teams that include attendings, fellows, and physician trainees.^23^ Inpatient team dynamics likely influence APP antibiotic decision-making. Surveys have found inpatient NPs are more likely to seek attending physician input on antimicrobial decisions compared to NPs practicing in community or outpatient settings.^24^ In addition, APPs practicing at one academic medical center reported “Attending or Fellow Decision” as the most common barrier to decreasing inappropriate antimicrobial prescriptions.^25^ Another study found that “fear of offending senior clinician” is a commonly cited factor in antibiotic decision-making among NPs.^26^

## How have ASPs historically engaged with APPs?

APPs have reported high motivation to be antibiotic stewards and have a high perception of the risks of antimicrobial overuse, such as resistance development and adverse effects.^24,27^ Taking these qualities and importance to patient care into account, there have long been calls for increased APP involvement with antimicrobial stewardship programs (ASPs).^28^ However, ASPs have not traditionally prioritized APP engagement in stewardship education and interventions, instead focusing on physician, physician trainees, pharmacists, and pharmacy trainees.^29,30^ It is not surprising that early studies found APPs did not have favorable perceptions of ASP interactions. One early study of NPs at a tertiary care medical center found a majority (66%) reported they were “not familiar” with the center’s ASP and only 17% reported finding ASP interactions “useful” or “very useful.”^27^ More recent studies suggest that ASP engagement of APPs is increasing with several more recent studies of APPs finding that the majority of NPs and/or PAs now report ASPs as helpful sources for ID and antimicrobial education were “somewhat useful or useful.”^24,25^

There have been few studies on ASP interventions that target APPs with the goal of improving antimicrobial prescribing, but those published found success in ambulatory settings. One of our authors’ (GMW) own experience was a study of four pediatric urgent care centers staffed by NPs and found a significant decrease in inappropriate prescribing for NPs that attended disease-specific educational sessions.^31^ An additional study found that the introduction of an acute otitis media bundle, including clinical decision support tools around guideline recommendations and appropriate dosing of antibiotic, to a pediatric hospital-owned retail clinic staffed by NPs and PAs improved appropriate antibiotic prescriptions and selections and, importantly, were sustained over the study follow-up (18 months).^32^

Studies on inpatient APP interventions have been limited by small study populations. We conducted a pilot study utilizing an educational intervention to decrease antimicrobial prescribing for asymptomatic bacteriuria among 33 inpatient Neurocritical Care and Cardiac Surgery NPs and PAs. We found that the educational intervention was well-received and improved knowledge acquisition; however, there was no change between pre- and post-intervention prescribing patterns for asymptomatic bacteriuria.^25^ Another study on NPs working on cardiothoracic surgery inpatient wards found success in increasing antimicrobial guideline adherence through targeted prescribing feedback coupled with educational outreach; however, the study only involved 11 NPs.^33^

Direct engagement of prescriber groups through education is recommended to supplement ASP activities; however, there may be unique barriers for APPs.^34^ A reported lack of protected time for education as well as perceptions that continuing education curricula were primarily designed for medical students and physician trainees are both factors that could lead to decreased APP engagement in ASP educational activities.^25,35^ We had also previously hypothesized that the culture at medical centers could be contributing to a lack of APP engagement, exemplified through the use of terms such as “nonteaching services,” “resident substitutes,” or “midlevels” that remain prevalent in both clinical and research settings and may perpetuate historical biases towards APPs.^5,36–39^

## Utilization of APPs on antimicrobial stewardship teams

There is a long history of APPs specializing in ID with the earliest reports of multi-disciplinary ambulatory HIV care teams coming from the late 1990s and inpatient ID consult teams from the mid-2000s.^40,41^ APPs in ID continue to grow with a survey of ID physicians and APPs reporting around 71% of ID physicians currently work with APPs in their practice.^42^ The role of ID APPs also continues to evolve with increased non-clinical, research, and leadership opportunities available, and, recently, 22% of ID APPs reported having antimicrobial stewardship roles at their medical center.^7,42^

We could not find further literature describing specific APPs roles in ASPs. However, we (authors GMW and JCH) have a long-standing history of APP team members on our ASP team. Our ASP APP is integrated into all aspects of our ASP program. For example, our dedicated ASP APP provides a portion of scheduled ASP coverage for our prospective audit with feedback service. This allows for the ASP pharmacists to have dedicated administrative time for other required activities, including formulary management requirements and data management of performance measure along with surveillance. This dedicated time also allows for professional development opportunities for our ASP pharmacists.

This relatively unique situation arose from inclusion of nursing representation in our ASP in the form of a program manager with a nursing background and a pediatric nurse practitioner early on in our program’s evolution. Our ASP APP’s role was initially sponsored and heavily supported by the organization’s Chief Nursing Officer. Capitalizing on the NP background of our ASP APP, we believe a valuable perspective is contributed, one that combines prescriber, nurse, and antimicrobial steward. Having this not only uncovered barriers impacting antimicrobial prescribing specific to APPs but additionally helped us design and implement initiatives that included nursing and APPs. These have included working to empower front-line nursing staff through education and support around obtaining timely cultures, IV to PO conversion, allergy/adverse reaction clarification and documentation, as well as a major undertaking to address antibiotic waste. These efforts have also led to APPs initiating ASP-related quality improvement work in diverse areas of the hospital. Specific examples of the clear benefits of APP involvement within our ASP are highlighted in Box 1.

Box 1. Examples of Successful Initiatives with APP Involvement

1. *Front-Line RN and Prescriber Engagement to Decrease Antimicrobial Waste*: nursing staff noted antibiotic doses being wasted due to the surgical teams not discontinuing the antibiotic order or extending the duration for a longer time than recommended with subsequent discharge of the patient from the hospital. Many of the antibiotics additionally had supply chain shortages. Our ASP team engaged not only the front-line RNs but surgical APPs, physicians, and our information technology department to standardize post-operative orders. This additionally led to the monitoring of other antimicrobial waste and highlighting the important role of stewardship in conservation and allocation of resources.
2. *Peer APP to APP Antimicrobial Stewardship Education and Outreach*: education is a key component for improving clinical practice among front-line APPs, and our ASP APP has allowed for expansion of educational access. For example, all onboarding APPs receive an ASP overview lecture by our ASP APP member. We feel that it is very beneficial for this group of providers to receive this education from a peer, similar to utilizing physicians and pharmacists for their peer groups. Additionally, when our ASP has undertaken new initiatives coupled with education to prescribers, our ASP APP member ensures these are communicated to APPs across the organization through appropriate channels and venues that are sometimes different than other clinical staff. These educational efforts serve an additional purpose of establishing relationships for future collaborations.

## Our thoughts on effective APP engagement by antimicrobial stewardship programs

Box 2. Where to Start? Recommendations for Antimicrobial Stewardship Programs (ASPs) and Stewards in Engaging Advanced Practice Providers (APPs)

- Identify key APP leadership at both division and institutional levels
- Include APP representation on ASP sub-committees and oversight committees, quality improvement projects, and guideline development workgroups
- Avoid “One Size Fits All” approach to stewardship interventions by recognizing APP unique characteristics and barriers to influencing antimicrobial prescribing
- Institute ASP-dedicated roles for both ID APPs and/or non-ID APPs
- Invest in educational resources, such as the Infectious Diseases Society of America Antimicrobial Stewardship Curriculum, for APPs in ID or stewardship roles

First, it is crucial to identify key APP leadership. For the majority of medical centers employing APPs, there is often a Director of Advanced Practice that can be engaged to help guide initiative development or dissemination of resources (guidelines, practice alerts).^43^ In addition, each division is likely to have an APP lead that oversees both inpatient and outpatient APPs. In our experience, these division leads can be key resources for ASPs. Furthermore, our own institutions have APP Councils that bring together APP leads in a multi-disciplinary forum to discuss practice-related issues, professional development opportunities, APP recognition, and promote overall collaboration and idea exchange among the divisions.

Second, we encourage ASPs to strongly consider APP representation on ASP sub-committees or oversight committees, quality improvement projects, and guideline development workgroups. For example, at one of our community hospitals, we invited APPs from the Critical Care group to join our ASP sub-committee. We found these Critical Care APPs provided a more longitudinal presence in the ICUs than attending physicians, who tended to rotate on service one week every one or two months. APP sub-committee membership also provided better dissemination of stewardship recommendations, particularly during the COVID-19 pandemic where recommendations evolved quickly. Similarly, inpatient APPs often develop a specialized clinical focus and should also be considered for guideline development workgroups (eg, colorectal surgery APPs and an intra-abdominal infection guideline). APP involvement would provide key group representation and perspective as well as a peer champion to aid with effective guideline dissemination and education.

Third, one of the tenets of antimicrobial stewardship interventions is customization to fit institutional needs and providers, and a similar approach should be taken when considering how to effectively engage APPs, avoiding a “One Size Fits All” approach.^34^ It is important to recognize the diversity within the term “Advanced Practice Provider.” For example, CRNAs have primary exposure to antimicrobials through surgical prophylaxis, while an NP on an inpatient orthopedic surgery team would be involved with antimicrobials for prosthetic joint infections. Interventions, particularly if involving educational components, should be tailored to fit the provider group’s background, interests, and mechanisms for communication/engagement. ASPs need to be cognizant that APPs may have different and unique barriers to effective interventions as outlined in the prior sections. Potential solutions would include working with APP leadership on local needs assessments and tailoring of educational sessions and materials, such as inclusion of self-directed modules and on-demand virtual components.^44^

Lastly, APPs, particularly those specializing in ID, could be considered for dedicated antimicrobial stewardship roles. We have outlined our authors’ own ASP experience in the prior section, but APPs have successfully been incorporated into inpatient ID consult teams and as sole primary providers in outpatient ID and outpatient antibiotic therapy (OPAT) clinics with benefits including expanding patients’ access to care and providing continuity of care.^41,45^ Depending on the institution’s program, ASP APP responsibilities could take the form of prior authorization of restricted antimicrobials (eg, antimicrobial approval pager coverage), assigned days for prospective audit and feedback, or providing peer education as an ID/ASP expert. An ASP APP could fill critical roles in expanding an ASPs’ reach, particularly in outpatient and emergency department settings that remain areas of need.^46,47^ These practice settings employ APPs at a higher rate than inpatient settings, and APPs could provide a critical perspective on intervention development and implementation.^2^ While there have not been published studies on the financial benefits of ASP utilization of APPs, APPs may help expand the pool of available stewards at a lower FTE rate than physicians or pharmacists, which may benefit smaller ASPs where the financial resources can be limited.

There has also been concern about the ongoing shortage of ID physicians, which would have a downstream effect of impacting ASP participation.^48^ A recent study found that while the presence of on-site ID specialists was associated with lower antimicrobial utilization, almost 15% of VA hospitals did not have on-site ID physicians – a number previously reported as even higher in smaller, community hospitals.^49–51^ Hospitalist APPs have been reported to fill key ASP roles at sites without on-site ID specialists, and this avenue should be continued to be explored.^52^ It is not clear whether APPs in stewardship roles would fulfill regulatory requirements if ID-trained physicians or pharmacists are not available. The most recent Center for Medicare and Medicaid Services (CMS) standards (482.42b) state that “the hospital must designate an individual or a group of individuals as its antibiotic stewardship program leaders” qualified through education, training, or certification.^53^ CMS does state that the “ideal” situation is a jointly led program led by a physician and pharmacist, but this may be difficult for smaller and rural community hospitals to achieve. In contrast, The Joint Commission requirement (EP11) does specify that the leader of the ASP is a “physician and/or pharmacist.”^54^

A lack of standardized ID-specific training has been reported as a perceived barrier to increasing APPs in ID, and a similar concern may arise in utilizing both ID and non-ID APPs in formal stewardship roles.^42^ However, the Infectious Diseases Society of America has created both an online Core and Advanced Antimicrobial Stewardship Curriculum. While these courses were initially designed and have already been found to be effective for ID fellows, the IDSA does specifically mention that other “stewards-in-training” groups, including APPs, can utilize and benefit from the curricula.^55,56^ For ID APPs, opportunities for involvement in ASPs might have the benefit of providing not only additional professional fulfillment but also non-clinical salary support to help potentially reduce risk of APP burnout and maintain a robust ID and stewardship workforce.

## Conclusion

With APPs taking on a more primary role in patient care in both the ambulatory and acute care settings, APPs are a prime group of healthcare providers that should not only be targeted for ASP interventions but also empowered to be front-line antimicrobial stewards and considered for dedicated ASP-related roles. APP inpatient antimicrobial prescribing practices, APP groups other than NPs and APPs, and outcomes related to APPs on ASPs have not been studied and remain important gaps that need to be further explored. Engaging APPs as well as their inclusion in ASP roles has the potential benefit to influence front-line antimicrobial prescribing as well as expand the scope and reach of stewardship programs.

## References

[1] U.S. Department of Health and Human Services, Health Resources and Services Administration, National Center for Health Workforce Analysis. Brief summary results from the 2018 National Sample Survey of Registered Nurses, 2019. https://bhw.hrsa.gov/sites/default/files/bureau-health-workforce/data-research/nssrn-summary-report.pdf. Published 2019. Accessed January 22, 2023

[2] American Association of Nurse Practitioners. American Association of Nurse Practitioners – NP Facts, 2022. https://www.aanp.org/about/all-about-nps/np-fact-sheet. Published 2022. Accessed January 15, 2023

[3] American Academy of Physician Assistants. What is a PA?, 2023. https://www.aapa.org/download/80021/. Published 2023. Accessed January 15, 2023

[4] Sym D , Brennan CW , Hart AM , Larson E. Characteristics of nurse practitioner curricula in the United States related to antimicrobial prescribing and resistance. J Am Acad Nurse Pract 2007;19:477–485. 10.1111/j.1745-7599.2007.00240.x 17760572

[5] Moote M , Krsek C , Kleinpell R , Todd B. Physician assistant and nurse practitioner utilization in academic medical centers. Am J Med Qual 2011;26:452–460. 10.1177/1062860611402984 21555487

[6] Sarzynski E , Barry H. Current evidence and controversies: advanced practice providers in healthcare. Am J Manag Care 2019;25:366–368. 31419093

[7] Kleinpell R , Cook ML , Padden DL. American association of nurse practitioners national nurse practitioner sample survey: update on acute care nurse practitioner practice. J Am Assoc Nurse Pract 2018;30:140–149. 10.1097/JXX.0000000000000030 29757882

[8] Kartha A , Restuccia JD , Burgess JF , et al. Nurse practitioner and physician assistant scope of practice in 118 acute care hospitals: NP and PA Scope of Practice. J Hosp Med 2014;9:615–620. 10.1002/jhm.2231 25224593

[9] Gail C , Field KW , Simpson T , Bond EF. Clinical nurse specialists and nurse practitioners: complementary roles for infectious disease and infection control. Am J Infect Control 2004;32:239–242. 10.1016/j.ajic.2003.06.002 15175622

[10] Sears A , Davis J , Zuber K. Postgraduate education and training for the nephrology physician assistants and nurse practitioners. Adv Chronic Kidney Dis 2022;29:534–538. 10.1053/j.ackd.2022.03.007 36371118

[11] Lerch W , Williams K , Polak C , Rometo A , Comunale MJ , Reynolds B. Establishment of pediatric subspecialty advanced practice provider fellowship training programs to optimize advanced practice utilization in pediatric specialty care and facilitate interprofessional integration. J Contin Educ Nurs 2022;53:478–480. 10.3928/00220124-20221006-02 36318714

[12] Sanchez GV , Hersh AL , Shapiro DJ , Cawley JF , Hicks LA. Outpatient antibiotic prescribing among United States nurse practitioners and physician assistants. Open Forum Infect Dis 2016;3:ofw168. 10.1093/ofid/ofw168 27704022 PMC5047413

[13] King LM , Bartoces M , Fleming-Dutra KE , Roberts RM , Hicks LA. Changes in US outpatient antibiotic prescriptions from 2011–2016. Clin Infect Dis. Published 2019. 10.1093/cid/ciz225 PMC8078491 30882145

[14] Eudy JL , Pallotta AM , Neuner EA , et al. Antimicrobial stewardship practice in the ambulatory setting from a national cohort. Open Forum Infect Dis 2020;7:ofaa513. 10.1093/ofid/ofaa513 33269298 PMC7686658

[15] Ladd E. The use of antibiotics for viral upper respiratory tract infections: an analysis of nurse practitioner and physician prescribing practices in ambulatory care, 1997–2001. J Am Acad Nurse Pract 2005;17:416–424. 10.1111/j.1745-7599.2005.00072.x 16181264

[16] Ference EH , Min JY , Chandra RK , et al. Antibiotic prescribing by physicians versus nurse practitioners for pediatric upper respiratory infections. Ann Otol Rhinol Laryngol 2016;125:982–991. 10.1177/0003489416668193 27707916

[17] Frost HM , McLean HQ , Chow BDW. Variability in antibiotic prescribing for upper respiratory illnesses by provider specialty. J Pediatr 2018;203:76–85.e8. 10.1016/j.jpeds.2018.07.044 30195553

[18] Schmidt ML , Spencer MD , Davidson LE . Patient, provider, and practice characteristics associated with inappropriate antimicrobial prescribing in ambulatory practices. Infect Control Hosp Epidemiol 2018;39:307–315. 10.1017/ice.2017.263 29378672

[19] Desai NM , Sadlowski JL , Mistry RD. Antibiotic prescribing for viral respiratory infections in the pediatric emergency department and urgent care. Pediatr Infect Dis J 2020;39:406–410. 10.1097/INF.0000000000002586 32176186

[20] O’Neill D , Branham S , Reimer A , Fitzpatrick J. Prescriptive practice differences between nurse practitioners and physicians in the treatment of uncomplicated urinary tract infections in the emergency department setting. J Am Assoc Nurse Pract 2021;33:194–199. 10.1097/JXX.0000000000000472 32976254

[21] Knobloch MJ , Musuuza J , Baubie K , Saban KL , Suda KJ , Safdar N. Nurse practitioners as antibiotic stewards: examining prescribing patterns and perceptions. Am J Infect Control 2021;49:1052–1057. 10.1016/j.ajic.2021.01.018 33524451 PMC8366016

[22] Fang FY , Weir TB , Codd CM , May CC , Abzug JM. Discharge opioid prescribing patterns in an academic orthopaedic setting: level of training and subspecialty patterns. J Am Acad Orthop Surg 2022;30:e361–e370. 10.5435/JAAOS-D-21-00895 34844260

[23] Chambers HE , Reinschmidt K , Smith G , et al. Examining the critical role of advanced practice providers on a multidisciplinary transplant team. Am J Transplant 2021;21:3840–3846. 10.1111/ajt.16715 34101989

[24] Hamilton RM , Merrill KC , Luthy KE , Nuttall C . Knowledge, attitudes, and perceptions of nurse practitioners about antibiotic stewardship. J Am Assoc Nurse Pract 2021;33:909–915. 10.1097/JXX.0000000000000467 32740336

[25] Lee MSL , Stead W. A seat at the table: delivering effective infectious diseases and antimicrobial stewardship education to advanced practice providers at an academic medical center. J Contin Educ Health Prof 2022;42:e27–e31. 10.1097/CEH.0000000000000383 34393185

[26] Smoke SM , Centrella M , Grigoriu A , Brust-Sisti L. Antibiotic decision-making among medical residents, medical students, and nurse practitioners: a single-center survey. J Pharm Pract 2019;32:372–374. 10.1177/0897190018821265 30595078

[27] Abbo L , Smith L , Pereyra M , Wyckoff M , Hooton TM. Nurse practitioners’ attitudes, perceptions, and knowledge about antimicrobial stewardship. J Nurse Pract 2012;8:370–376. 10.1016/j.nurpra.2012.01.023

[28] Manning ML. The urgent need for nurse practitioners to lead antimicrobial stewardship in ambulatory health care. J Am Assoc Nurse Pract 2014;26:411–413. 10.1002/2327-6924.12150 25048218

[29] Silverberg SL , Zannella VE , Countryman D , et al. A review of antimicrobial stewardship training in medical education. Int J Med Educ 2017;8:353–374. 10.5116/ijme.59ba.2d47 29035872 PMC5694692

[30] Ramakrishnan A , Patel PK. How far we’ve come, how far we have to go: a review of advances in antimicrobial stewardship in the veterans health administration. Curr Treat Options Infect Dis 2020;12:275–284. 10.1007/s40506-020-00221-w 33244296 PMC7688065

[31] Weddle G , Goldman J , Myers A , Newland J. Impact of an educational intervention to improve antibiotic prescribing for nurse practitioners in a pediatric urgent care center. J Pediatr Health Care 2017;31:184–188. 10.1016/j.pedhc.2016.07.005 27567148

[32] Joo KR , Sandberg K , Albertini B , Sowar L , Ziemnik LS. QI project promoting NP compliance with an AOM bundle in pediatric hospital-owned retail clinic. Pediatr Qual Saf 2022;7:e537. 10.1097/pq9.0000000000000537 35369407 PMC8970111

[33] Inkster T , Marek A , Khanna N. Improving antimicrobial prescribing by targeting clinical nurse practitioners. J Hosp Infect 2010;76:85–86. 10.1016/j.jhin.2010.05.009 20663586

[34] Barlam TF , Cosgrove SE , Abbo LM , et al. Implementing an antibiotic stewardship program: guidelines by the infectious diseases society of America and the society for healthcare epidemiology of America. Clin Infect Dis 2016;62:e51–e77. 10.1093/cid/ciw118 27080992 PMC5006285

[35] McGrath BA , Jacobs ML , Watts RM , Callender BC. Developing and sustaining advanced practice provider services: a decade of lessons learned. J Hosp Med 2022;17:1014–1020. 10.1002/jhm.12932 35942984 PMC10087730

[36] Reines HD , Robinson L , Duggan M , O’Brien BM , Aulenbach K. Integrating midlevel practitioners into a teaching service. Am J Surg 2006;192:119–124. 10.1016/j.amjsurg.2006.01.047 16769288

[37] Hoyt KS. Why the terms “Mid-Level Provider” and “Physician Extender” are inappropriate. Adv Emerg Nurs J 2012;34:93–94. 10.1097/TME.0b013e3182532114 22561218

[38] Repp AB , Bartsch JC , Pasanen ME. What the “Nonteaching” service can teach us. Acad Med 2018;93:41–44. 10.1097/ACM.0000000000001833 28746070

[39] Steward WT , Koester KA , Guzé MA , et al. Practice transformations to optimize the delivery of HIV primary care in community healthcare settings in the United States: a program implementation study. Rosen S, ed. PLOS Med 2020;17:e1003079. 10.1371/journal.pmed.1003079 32214312 PMC7098549

[40] Davis S. The role of advanced practice in today’s infectious diseases. Crit Care Nurs Q 1999;21:22–30. 10.1097/00002727-199902000-00005 10646429

[41] White CN , Borchardt RA , Mabry ML , Smith KM , Mulanovich VE , Rolston KV. Multidisciplinary cancer care: development of an infectious diseases physician assistant workforce at a comprehensive cancer center. J Oncol Pract 2010;6:e31–e34. 10.1200/JOP.2010.000100 21358948 PMC2988676

[42] Beieler AM , Yoke LH , Liu C , Pergam SA , Wald A , Dhanireddy S. Advanced practice providers in the infectious disease workforce: a nationwide utilization survey. J Interprofessional Educ Pract 2021;24:100448. 10.1016/j.xjep.2021.100448 PMC9765300 36567810

[43] Torbeck LJ , Hockaday M , Smith C , Dunnington G. Optimizing the integration of advanced practitioners in a department of surgery: an operational improvement model. Am J Surg 2019;217:597–604. 10.1016/j.amjsurg.2018.07.013 30055805

[44] Yoke LH , Beieler AM , Liu C , Pergam SA , Dhanireddy S. Advanced practice providers in infectious disease: educational needs and opportunities. Open Forum Infect Dis 8:S574–S575.

[45] Klemmer D , Shand W , Zibell C. Description of nurse practitioner functioning as most responsible provider in an intravenous therapy clinic. J Am Assoc Nurse Pract 2022. Publish Ahead of Print. 10.1097/JXX.0000000000000752 35727193

[46] Marcelin JR , Chung P , Van Schooneveld TC. Antimicrobial stewardship in the outpatient setting: a review and proposed framework. Infect Control Hosp Epidemiol 2020;41:833–840. 10.1017/ice.2020.94 32342826

[47] El Feghaly RE , Monsees EA , Burns A , et al. Outpatient antimicrobial stewardship programs in pediatric institutions in 2020: status, needs, barriers. Infect Control Hosp Epidemiol 2022;43:1396–1402. 10.1017/ice.2021.416 34674785

[48] del Rio C. IDSA Statement on ID Fellowship Match Rates; 2022. https://www.idsociety.org/news--publications-new/articles/2022/idsa-statement-on-id-fellowship-match-rates/. Accessed January 27, 2023

[49] Livorsi DJ , Nair R , Lund BC , et al. Antibiotic stewardship implementation and antibiotic use at hospitals with and without on-site infectious disease specialists. Clin Infect Dis 2021;72:1810–1817. 10.1093/cid/ciaa388 32267496

[50] Septimus EJ , Owens RC. Need and potential of antimicrobial stewardship in community hospitals. Clin Infect Dis 2011;53(suppl_1):S8–S14. 10.1093/cid/cir363 21795728

[51] Vaughn VM , Greene MT , Ratz D , et al. Antibiotic stewardship teams and *Clostridioides difficile* practices in United States hospitals: a national survey in The Joint Commission antibiotic stewardship standard era. Infect Control Hosp Epidemiol 2019:1–6. Published 2019. 10.1017/ice.2019.313 31806059

[52] Livorsi DJ , Stewart Steffensmeier KR , Perencevich EN , Goetz MB , Schacht Reisinger H. Antibiotic stewardship implementation at hospitals without on-site infectious disease specialists: a qualitative study. Infect Control Hosp Epidemiol 2022;43:576–581. 10.1017/ice.2021.203 33993897 PMC9096709

[53] Center for Medicare and Medicaid Services. Infection prevention and control and antibiotic stewardship program interpretive guidance update, 2022. https://www.cms.gov/files/document/qso-22-20-hospitals.pdf. Published 2022. Accessed April 11, 2023

[54] The Joint Commission. R3 Report – New and Revised Requirements for Antibiotic Stewardship; 2022. https://www.jointcommission.org/-/media/tjc/documents/standards/r3-reports/r3_antibioticstewardship_july2022_final.pdf. Published 2022. Accessed April 11, 2023

[55] Spicer JO , Armstrong WS , Schwartz BS , et al. Evaluation of the infectious diseases society of America’s core antimicrobial stewardship curriculum for infectious diseases fellows. Clin Infect Dis 2022;74:965–972. 10.1093/cid/ciab600 34192322

[56] Infectious Diseases Society of America. IDSA core antimicrobial stewardship curriculum fellow/trainee roadmap. https://academy.idsociety.org/sites/default/files/2019%20CAS%20Curriculum_Trainee%20User%20Guide_0.pdf. Accessed February 1, 2023

